# Mapping the exposome of mental health: exposome-wide association study of mental health outcomes among UK Biobank participants

**DOI:** 10.1192/j.eurpsy.2025.353

**Published:** 2025-08-26

**Authors:** A. Arias, L.-K. Pries, B. D. Lin, S. Guloksuz

**Affiliations:** 1 Department of Psychiatry and Neuropsychology, School for Mental Health and Neuroscience, Maastricht University Medical Centre, Maastricht, Netherlands; 2 Department of Preventive Medicine, Institute of Biomedical Informatics, Bioinformatics Center, School of Basic Medical Sciences, Henan University, Kaifeng, China; 3 Department of Psychiatry, Yale University School of Medicine, New Haven, United States

## Abstract

**Introduction:**

Mental disorders result from a complex interplay of genetic and environmental factors. However, the multiplicity of exposures and the complexity of mental health phenotypes pose a major challenge. The ‘exposome’ paradigm offers a holistic view of the environment that contrasts traditional hypothesis-driven approach in psychiatry. Within this framework, exposome-wide studies provide a novel tool to systematically identify phenotype-exposure relationships, offering an innovative perspective to map the exposome of mental health.

**Objectives:**

To map environmental factors associated with psychiatric diagnostic domains and symptom dimensions in the UK Biobank cohort. In this study, we aim to identify exposures unique to specific mental health outcomes, as well as those shared across conditions.

**Methods:**

We analysed UK Biobank participants with complete Mental Health Questionnaire data (N = 157,298). Outcomes were classified as either psychiatric domains or symptom dimensions. After quality control, 294 environmental, lifestyle, behavioral, and economic variables were included. An Exposome-Wide Association Study (ExWAS) was conducted per outcome in two equally split datasets, applying Bonferroni correction for multiple testing (P < 1.70 x 10^-4). Missing exposure data was imputed using Multiple Imputation by Chained Equations. Variables associated with each outcome were then tested in a multivariable model.

**Results:**

In diagnostic domains, ExWAS analyses identified 26 to 165 significant factors. Multivariable analysis revealed 10 to 65 significant associations, with traumatic events, physical complaints, and sleep disturbances emerging across domains. Cannabis use was associated with common psychiatric disorders (ORs: 1.10-1.79), while computer use was uniquely linked to neurodevelopmental disorders (OR = 1.23). Eating disorders showed stronger correlations with food-related exposures. In symptom dimensions, ExWAS identified 46 to 180 significant factors. Multivariable analysis revealed similar exposure groups to those in diagnostic domains. Notably, self-harm was uniquely associated with childhood adoption (OR = 1.39).

**Conclusions:**

This comprehensive mapping of exposome revealed that several factors, particularly in the domains of those previously well-studied were shared across mental health phenotypes, providing further support for transdiagnostic pathoetiology. Our findings also showed that distinct relations might exist. Continued exposome research through multimodal mechanistic studies guided by the transdiagnostic mental health framework is required to better inform public health policies.

**Disclosure of Interest:**

None Declared

